# 4-Bromo­anilinium hexa­fluoro­phosphate monohydrate

**DOI:** 10.1107/S1600536810018088

**Published:** 2010-05-22

**Authors:** Yong-le Yang, Xue-qun Fu

**Affiliations:** a Ordered Matter Science Research Center, Southeast University, Nanjing 210096, People’s Republic of China

## Abstract

In the title compound, C_6_H_7_BrN^+^·PF_6_^−^·H_2_O, N—H⋯F, N—H⋯O and O—H⋯F hydrogen-bonding inter­actions stabilize the crystal structure and give rise to to chains running parallel to the *c* axis. In the anion, four of the F atoms are disordered over two sets of sites of equal occupancy.

## Related literature

The title compound was synthesized as part of our group’s search for ferroelectric compounds, which usually have a phase transition. For background to phase transition materials, see: Li *et al.* (2008 ▶); Zhang *et al.* (2009 ▶).
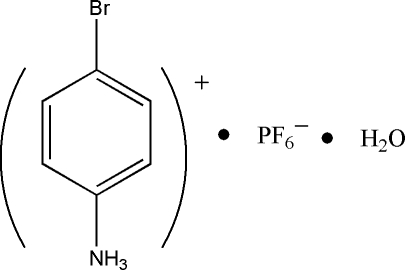


## Experimental

### 

#### Crystal data

C_6_H_7_BrN^+^·PF_6_^−^·H_2_O
*M* *_r_* = 336.02
Monoclinic, 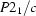

*a* = 14.646 (8) Å
*b* = 5.075 (3) Å
*c* = 15.314 (8) Å
β = 94.697 (11)°
*V* = 1134.5 (10) Å^3^
*Z* = 4
Mo *K*α radiation
μ = 3.82 mm^−1^
*T* = 298 K
0.20 × 0.20 × 0.20 mm

#### Data collection

Rigaku SCXmini diffractometer
Absorption correction: multi-scan (*CrystalClear*; Rigaku, 2005 ▶) *T*_min_ = 0.465, *T*_max_ = 0.484
11563 measured reflections
2584 independent reflections
1949 reflections with *I* > 2σ(*I*)
*R*_int_ = 0.043

#### Refinement

*R*[*F* ^2^ > 2σ(*F* ^2^)] = 0.051
*wR*(*F* ^2^) = 0.143
*S* = 1.05
2584 reflections
189 parameters
24 restraints
H atoms treated by a mixture of independent and constrained refinement
Δρ_max_ = 0.48 e Å^−3^
Δρ_min_ = −0.54 e Å^−3^

### 

Data collection: *CrystalClear* (Rigaku, 2005 ▶); cell refinement: *CrystalClear*; data reduction: *CrystalClear*; program(s) used to solve structure: *SHELXS97* (Sheldrick, 2008 ▶); program(s) used to refine structure: *SHELXL97* (Sheldrick, 2008 ▶); molecular graphics: *SHELXTL* (Sheldrick, 2008 ▶); software used to prepare material for publication: *PRPKAPPA* (Ferguson, 1999 ▶).

## Supplementary Material

Crystal structure: contains datablocks I, global. DOI: 10.1107/S1600536810018088/jh2158sup1.cif

Structure factors: contains datablocks I. DOI: 10.1107/S1600536810018088/jh2158Isup2.hkl

Additional supplementary materials:  crystallographic information; 3D view; checkCIF report

## Figures and Tables

**Table 1:** Hydrogen-bond geometry (Å, °)

*D*—H⋯*A*	*D*—H	H⋯*A*	*D*⋯*A*	*D*—H⋯*A*
N1—H1*B*⋯F6	0.89	2.59	3.13 (3)	120
N1—H1*C*⋯O1*W*	0.89	2.24	2.891 (5)	130
N1—H1*A*⋯F2^i^	0.89	2.15	3.02 (2)	168
N1—H1*B*⋯O1*W*^ii^	0.89	2.42	2.905 (5)	115
N1—H1*B*⋯F4^iii^	0.89	2.45	3.252 (5)	149
N1—H1*C*⋯F5^iv^	0.89	2.51	3.04 (2)	119
O1*W*—H1*WB*⋯F6^v^	0.75 (7)	2.34 (8)	3.00 (3)	148 (6)
O1*W*—H1*WB*⋯F1^v^	0.75 (7)	2.48 (7)	3.062 (5)	136 (6)
O1*W*—H1*WA*⋯F5^vi^	0.62 (7)	2.36 (7)	2.92 (2)	151 (7)

Symmetry codes: (i) 
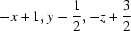
; (ii) 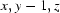; (iii) 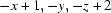; (iv) 
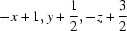
; (v) 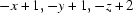; (vi) 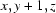.

**Fig. 1.**
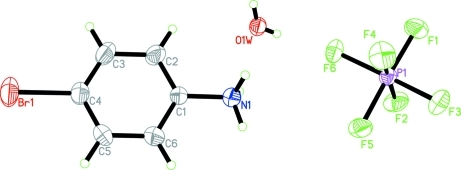
The molecular structure of the title compound, with the atomic numbering scheme. Displacement ellipsoids are drawn at the 30% probability level, and all H atoms have been omitted for clarity.

**Fig. 2.**
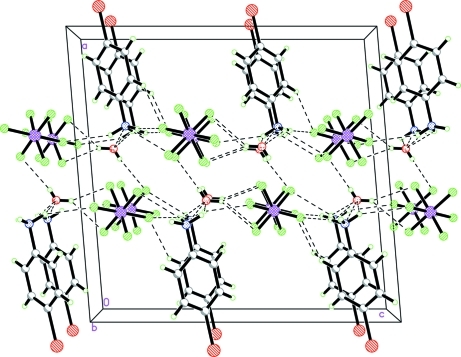
A view of the packing of the title compound, stacking along the b axis. Dashed lines indicate hydrogen bonds.

## supplementary crystallographic information

### Comment

As a continuation of our study of phase transition materials, including organic ligands (Li *et al.*, 2008), metal-organic coordination compounds (Zhang *et al.*, 2009 ), organic-inorganic hybrids, we studied the dielectric properties of the title compound, unfortunately, there was no distinct anomaly observed from 93 K to 350 K,suggesting that this compound should be not a real ferroelectrics or there may be no distinct phase transition occurred within the measured temperature range. In this article, the crystal structure of (I) has been presented.

The asymmetric unit of the title compound is made up of a almost coplanar 4-bromoanilimium cation with the mean deviation from the plan of 0.013 Å, a hexafluorophosphate anion disordered intwo orientations with site-occupancy factors of 0.7365 and 0.2635, and a water molecule. The chains of the molecular arrangement in the crystal structure is mainly determined by relatively strong and directional N—H···F, N—H···O and O—H···F hydrogen bonds (Table 1), and to a lesser degree by a π-π packing interaction between the adjacent aromatic rings, where the interplanar spacing is 5.855 (4) Å.

### Experimental

Single crystals of 4-bromoanilimium hexafluorophosphate monohydrate were prepared by slow evaporation at room temperature of an ethanol solution of equal molar 4-bromobenzenamine and hexafluorophosphoric acid.

### Refinement

Positional parameters of all the H atoms were calculated geometrically and were allowed to ride on the C and N atoms to which they are bonded, with *U*_iso_(H) = 1.2U_eq_(C),

*U*_iso_(H) = 1.2U_eq_(N).

### Crystal data

**Table:**

C_6_H_7_BrN^+^·PF_6_^−^·H_2_O	*F*(000) = 656
*M**_r_* = 336.02	*D*_x_ = 1.967 Mg m^−^^3^
Monoclinic, *P*2_1_/*c*	Mo *K*α radiation, λ = 0.71073 Å
Hall symbol: -P 2ybc	Cell parameters from 2551 reflections
*a* = 14.646 (8) Å	θ = 3.7–27.5°
*b* = 5.075 (3) Å	µ = 3.82 mm^−^^1^
*c* = 15.314 (8) Å	*T* = 298 K
β = 94.697 (11)°	Prism, colorless
*V* = 1134.5 (10) Å^3^	0.20 × 0.20 × 0.20 mm
*Z* = 4	

### Data collection

**Table:**

Rigaku SCXmini diffractometer	2584 independent reflections
Radiation source: fine-focus sealed tube	1949 reflections with *I* > 2σ(*I*)
graphite	*R*_int_ = 0.043
Detector resolution: 13.6612 pixels mm^-1^	θ_max_ = 27.4°, θ_min_ = 3.1°
ω scans	*h* = −18→18
Absorption correction: multi-scan (*CrystalClear*; Rigaku, 2005)	*k* = −6→6
*T*_min_ = 0.465, *T*_max_ = 0.484	*l* = −19→19
11563 measured reflections	

### Refinement

**Table:**

Refinement on *F*^2^	Primary atom site location: structure-invariant direct methods
Least-squares matrix: full	Secondary atom site location: difference Fourier map
*R*[*F*^2^ > 2σ(*F*^2^)] = 0.051	Hydrogen site location: inferred from neighbouring sites
*wR*(*F*^2^) = 0.143	H atoms treated by a mixture of independent and constrained refinement
*S* = 1.05	*w* = 1/[σ^2^(*F*_o_^2^) + (0.0729*P*)^2^ + 0.7365*P*] where *P* = (*F*_o_^2^ + 2*F*_c_^2^)/3
2584 reflections	(Δ/σ)_max_ < 0.001
189 parameters	Δρ_max_ = 0.48 e Å^−^^3^
24 restraints	Δρ_min_ = −0.53 e Å^−^^3^

### Special details

**Table:**

Geometry. All esds (except the esd in the dihedral angle between two l.s. planes) are estimated using the full covariance matrix. The cell esds are taken into account individually in the estimation of esds in distances, angles and torsion angles; correlations between esds in cell parameters are only used when they are defined by crystal symmetry. An approximate (isotropic) treatment of cell esds is used for estimating esds involving l.s. planes.
Refinement. Refinement of *F*^2^ against ALL reflections. The weighted *R*-factor wR and goodness of fit *S* are based on *F*^2^, conventional *R*-factors *R* are based on *F*, with *F* set to zero for negative *F*^2^. The threshold expression of *F*^2^ > σ(*F*^2^) is used only for calculating *R*-factors(gt) etc. and is not relevant to the choice of reflections for refinement. *R*-factors based on *F*^2^ are statistically about twice as large as those based on *F*, and *R*- factors based on ALL data will be even larger.

### Fractional atomic coordinates and isotropic or equivalent isotropic displacement parameters (Å^2^)

**Table:**

	*x*	*y*	*z*	*U*_iso_*/*U*_eq_	Occ. (<1)
Br1	−0.07352 (3)	0.12750 (14)	0.89407 (4)	0.0887 (3)	
C1	0.2358 (2)	0.1568 (7)	0.8526 (2)	0.0410 (8)	
C2	0.2022 (3)	0.3220 (9)	0.9125 (3)	0.0690 (13)	
H2A	0.2410	0.4372	0.9449	0.083*	
C3	0.1092 (4)	0.3161 (11)	0.9246 (4)	0.0780 (15)	
H3A	0.0850	0.4279	0.9650	0.094*	
C4	0.0536 (3)	0.1443 (9)	0.8766 (3)	0.0564 (10)	
C5	0.0873 (3)	−0.0174 (13)	0.8167 (4)	0.0836 (16)	
H5A	0.0485	−0.1317	0.7840	0.100*	
C6	0.1802 (3)	−0.0121 (11)	0.8043 (4)	0.0770 (15)	
H6A	0.2041	−0.1228	0.7634	0.092*	
N1	0.3347 (2)	0.1574 (6)	0.8399 (2)	0.0449 (7)	
H1A	0.3460	0.0403	0.7989	0.067*	
H1B	0.3664	0.1151	0.8900	0.067*	
H1C	0.3513	0.3171	0.8231	0.067*	
O1W	0.4090 (2)	0.6558 (6)	0.9035 (3)	0.0497 (7)	
H1WB	0.407 (5)	0.646 (12)	0.952 (5)	0.10 (3)*	
H1WA	0.445 (5)	0.652 (12)	0.885 (4)	0.09 (3)*	
P1	0.62950 (6)	0.0473 (2)	0.87429 (6)	0.0426 (3)	
F1	0.69905 (19)	0.2001 (6)	0.94031 (19)	0.0747 (8)	
F2	0.6508 (16)	0.215 (4)	0.7908 (13)	0.069 (3)	0.50
F3	0.706 (2)	−0.161 (5)	0.852 (2)	0.067 (4)	0.50
F4	0.6103 (2)	−0.1569 (5)	0.95004 (19)	0.0753 (8)	
F5	0.5602 (14)	−0.141 (7)	0.8100 (14)	0.066 (5)	0.50
F6	0.541 (2)	0.203 (7)	0.908 (2)	0.065 (5)	0.50
F3'	0.7131 (19)	−0.097 (5)	0.8358 (18)	0.071 (5)	0.50
F6'	0.554 (2)	0.239 (7)	0.900 (2)	0.073 (6)	0.50
F2'	0.6400 (18)	0.284 (4)	0.8075 (12)	0.073 (3)	0.50
F5'	0.5575 (14)	−0.078 (7)	0.8065 (14)	0.071 (6)	0.50

### Atomic displacement parameters (Å^2^)

**Table:**

	*U*^11^	*U*^22^	*U*^33^	*U*^12^	*U*^13^	*U*^23^
Br1	0.0451 (3)	0.1472 (7)	0.0747 (4)	0.0010 (3)	0.0107 (2)	0.0271 (3)
C1	0.0418 (18)	0.0409 (19)	0.0404 (18)	0.0011 (15)	0.0029 (14)	0.0040 (15)
C2	0.063 (3)	0.069 (3)	0.079 (3)	−0.017 (2)	0.025 (2)	−0.029 (2)
C3	0.064 (3)	0.087 (4)	0.087 (4)	−0.001 (3)	0.031 (3)	−0.026 (3)
C4	0.044 (2)	0.079 (3)	0.046 (2)	0.004 (2)	0.0037 (17)	0.017 (2)
C5	0.047 (2)	0.117 (4)	0.086 (3)	−0.016 (3)	0.000 (2)	−0.039 (3)
C6	0.054 (3)	0.094 (4)	0.082 (3)	−0.003 (3)	0.001 (2)	−0.041 (3)
N1	0.0452 (16)	0.0415 (16)	0.0483 (17)	−0.0010 (13)	0.0057 (13)	0.0024 (14)
O1W	0.0439 (16)	0.0496 (17)	0.056 (2)	−0.0011 (13)	0.0094 (14)	−0.0043 (14)
P1	0.0394 (5)	0.0469 (5)	0.0426 (5)	−0.0052 (4)	0.0087 (4)	−0.0044 (4)
F1	0.0653 (16)	0.0864 (18)	0.0714 (17)	−0.0209 (14)	−0.0014 (13)	−0.0273 (15)
F2	0.085 (6)	0.075 (10)	0.051 (6)	−0.017 (6)	0.028 (4)	−0.002 (5)
F3	0.062 (8)	0.063 (9)	0.079 (8)	0.011 (6)	0.026 (5)	−0.006 (5)
F4	0.0805 (19)	0.0728 (17)	0.0732 (18)	−0.0039 (14)	0.0106 (14)	0.0252 (14)
F5	0.065 (5)	0.073 (13)	0.064 (5)	−0.030 (5)	0.021 (5)	−0.023 (5)
F6	0.045 (5)	0.077 (10)	0.075 (7)	0.008 (5)	0.018 (5)	−0.005 (7)
F3'	0.045 (4)	0.086 (13)	0.085 (11)	−0.005 (7)	0.026 (6)	−0.027 (8)
F6'	0.066 (11)	0.064 (8)	0.093 (9)	0.026 (9)	0.024 (7)	0.007 (5)
F2'	0.109 (8)	0.059 (8)	0.054 (7)	−0.013 (6)	0.027 (5)	0.007 (5)
F5'	0.060 (5)	0.077 (14)	0.072 (5)	−0.024 (5)	−0.015 (5)	−0.017 (5)

### Geometric parameters (Å, °)

**Table:**

Br1—C4	1.904 (4)	N1—H1C	0.8900
C1—C6	1.359 (6)	O1W—H1WB	0.75 (7)
C1—C2	1.364 (6)	O1W—H1WA	0.62 (7)
C1—N1	1.477 (5)	P1—F6'	1.55 (3)
C2—C3	1.390 (7)	P1—F5'	1.55 (2)
C2—H2A	0.9300	P1—F3'	1.58 (3)
C3—C4	1.366 (7)	P1—F1	1.579 (3)
C3—H3A	0.9300	P1—F2'	1.59 (2)
C4—C5	1.353 (7)	P1—F2	1.59 (2)
C5—C6	1.390 (7)	P1—F4	1.597 (3)
C5—H5A	0.9300	P1—F3	1.60 (3)
C6—H6A	0.9300	P1—F6	1.64 (3)
N1—H1A	0.8900	P1—F5	1.66 (2)
N1—H1B	0.8900		
			
C6—C1—C2	121.4 (4)	F6'—P1—F2	93.1 (15)
C6—C1—N1	118.6 (4)	F5'—P1—F2	81.3 (13)
C2—C1—N1	120.0 (3)	F3'—P1—F2	75.0 (10)
C1—C2—C3	119.2 (4)	F1—P1—F2	95.3 (8)
C1—C2—H2A	120.4	F2'—P1—F2	16.8 (8)
C3—C2—H2A	120.4	F6'—P1—F4	93.1 (14)
C4—C3—C2	119.3 (4)	F5'—P1—F4	94.1 (11)
C4—C3—H3A	120.3	F3'—P1—F4	99.0 (8)
C2—C3—H3A	120.3	F1—P1—F4	89.78 (17)
C5—C4—C3	121.1 (4)	F2'—P1—F4	170.9 (5)
C5—C4—Br1	118.9 (4)	F2—P1—F4	172.1 (5)
C3—C4—Br1	119.9 (3)	F6'—P1—F3	177.2 (16)
C4—C5—C6	119.8 (4)	F5'—P1—F3	92.0 (16)
C4—C5—H5A	120.1	F3'—P1—F3	15.2 (13)
C6—C5—H5A	120.1	F1—P1—F3	92.0 (12)
C1—C6—C5	119.1 (4)	F2'—P1—F3	104.6 (9)
C1—C6—H6A	120.4	F2—P1—F3	89.6 (10)
C5—C6—H6A	120.4	F4—P1—F3	84.1 (7)
C1—N1—H1A	109.5	F6'—P1—F6	10 (3)
C1—N1—H1B	109.5	F5'—P1—F6	84.2 (15)
H1A—N1—H1B	109.5	F3'—P1—F6	176.7 (16)
C1—N1—H1C	109.5	F1—P1—F6	92.5 (11)
H1A—N1—H1C	109.5	F2'—P1—F6	87.8 (15)
H1B—N1—H1C	109.5	F2—P1—F6	102.1 (15)
H1WB—O1W—H1WA	124 (8)	F4—P1—F6	83.7 (13)
F6'—P1—F5'	87.9 (17)	F3—P1—F6	167.0 (15)
F6'—P1—F3'	167.6 (15)	F6'—P1—F5	95.4 (16)
F5'—P1—F3'	93.7 (15)	F5'—P1—F5	11 (2)
F6'—P1—F1	88.2 (14)	F3'—P1—F5	88.1 (14)
F5'—P1—F1	174.7 (13)	F1—P1—F5	174.3 (12)
F3'—P1—F1	89.3 (11)	F2'—P1—F5	97.9 (13)
F6'—P1—F2'	78.2 (15)	F2—P1—F5	89.0 (12)
F5'—P1—F2'	88.5 (15)	F4—P1—F5	85.6 (10)
F3'—P1—F2'	89.5 (10)	F3—P1—F5	84.2 (15)
F1—P1—F2'	87.2 (9)	F6—P1—F5	90.3 (14)
			
C6—C1—C2—C3	0.3 (8)	C3—C4—C5—C6	0.8 (9)
N1—C1—C2—C3	−179.0 (4)	Br1—C4—C5—C6	−178.7 (5)
C1—C2—C3—C4	0.3 (8)	C2—C1—C6—C5	−0.4 (8)
C2—C3—C4—C5	−0.9 (8)	N1—C1—C6—C5	179.0 (5)
C2—C3—C4—Br1	178.6 (4)	C4—C5—C6—C1	−0.1 (9)

### Hydrogen-bond geometry (Å, °)

**Table:**

*D*—H···*A*	*D*—H	H···*A*	*D*···*A*	*D*—H···*A*
N1—H1B···F6	0.89	2.59	3.13 (3)	120
N1—H1C···O1W	0.89	2.24	2.891 (5)	130
N1—H1A···F2^i^	0.89	2.15	3.02 (2)	168
N1—H1B···O1W^ii^	0.89	2.42	2.905 (5)	115
N1—H1B···F4^iii^	0.89	2.45	3.252 (5)	149
N1—H1C···F5^iv^	0.89	2.51	3.04 (2)	119
O1W—H1WB···F6^v^	0.75 (7)	2.34 (8)	3.00 (3)	148 (6)
O1W—H1WB···F1^v^	0.75 (7)	2.48 (7)	3.062 (5)	136 (6)
O1W—H1WA···F5^vi^	0.62 (7)	2.36 (7)	2.92 (2)	151 (7)

Symmetry codes: (i) −*x*+1, *y*−1/2, −*z*+3/2; (ii) *x*, *y*−1, *z*; (iii) −*x*+1, −*y*, −*z*+2; (iv) −*x*+1, *y*+1/2, −*z*+3/2; (v) −*x*+1, −*y*+1, −*z*+2; (vi) *x*, *y*+1, *z*.
